# Gut microbiome alterations and their clinical and biological implications in ovarian cancer: a systematic review

**DOI:** 10.3389/fonc.2025.1690541

**Published:** 2025-11-28

**Authors:** Beibei Zhang, Nur Fatin Nabilah Mohd Sahardi, Kah Teik Chew, Wen Di, Mohamad Nasir Shafiee

**Affiliations:** 1 Department of Obstetrics and Gynecology, Faculty of Medicine, Universiti Kebangsaan Malaysia, Kuala Lumpur, Malaysia; 2 Faculty of Medicine, Universiti Kebangsaan Malaysia, Kuala Lumpur, Malaysia; 3 Department of Obstetrics and Gynecology, Renji Hospital, School of Medicine, Shanghai Jiao Tong University, Shanghai, China

**Keywords:** gut microbiome, ovarian cancer, cancer biomarkers, microbiome-based therapies, dysbiosis

## Abstract

**Background:**

Increasing evidence shows the that gut microbiome (GM) plays a crucial role in ovarian cancer (OC) progression, offering potential avenues for microbiome-based intervention strategies. However, research in this area remains limited. This systematic review aimed to synthesize current evidence on microbiome composition and diversity in OC, focusing on its association with disease diagnosis, postoperative changes, and responses to chemotherapy or PARP inhibitor (PARPi) therapy.

**Methods:**

A literature search was performed in PubMed and Web of Science up to October 2025 using keywords: (gut microb* OR gut bacteri* OR intestinal microb* OR intestinal bacteri*) AND (ovarian cancer OR ovarian carcinoma OR carcinoma of ovary). Only original research articles involving human subjects were included. Data on GM alterations in OC patients, postoperative changes, and responses to chemotherapy or PARP inhibitor (PARPi) therapy were extracted and analysed.

**Results:**

Nine eligible studies, comprising longitudinal and case-control studies were reviewed. At diagnosis, OC patients displayed gut dysbiosis characterised by an increase in Proteobacteria and a decrease in Firmicutes. Genus-level analysis revealed lower levels of *Akkermansia* and elevated levels of *Bacteroides* and *Prevotella*, suggesting disrupted microbial homeostasis. Following surgery, both Firmicutes and Proteobacteria declined, indicating significant microbiome shifts. During chemotherapy, especially neoadjuvant treatment, Firmicutes re-emerged as the dominant phylum. Family-level analyses identified increased Coriobacteriaceae and decreased Ruminococcaceae. Platinum-sensitive patients demonstrated more stable GM profiles than those with platinum resistance Genera such as *Angelakisella, Arenimonas, and Roseburia* emerged as potential candidates for diagnostic or prognostic markers of chemotherapy resistance. Meanwhile, *Phascolarbacterium* is identified as a PARPi response in BRCA1/2-negative OC, with higher levels linked to longer progression-free survival.

**Conclusion:**

This review highlights a dynamic GM composition in OC across disease stages and treatments, underscoring the need for further research on microbiome-targeted therapeutic strategies.

## 1 Introduction

Ovarian cancer (OC) is among the most common malignancies worldwide. Although it ranks third in incidence among gynecological cancers, following cervix and uterine cancers, it is the second leading cause of cancer-related mortality in women. According to cancer statistics published in 2022, OC accounts for 324,402 new cases and 206,839 deaths annually (1). Meanwhile, the number of women diagnosed with ovarian cancer is projected to exceed 503,448 by 2050 (1).

OC comprises several subtypes, with epithelial ovarian cancer (EOC) being the most prevalent, accounting for over 90% of cases, followed by germ cell tumors and sex cord-stromal tumors (2). EOC is widely considered a heterogeneous disease. Therefore, it has been classified into five main subtypes based on their clinicopathological features, which are high-grade serous ovarian cancer (HGSOC), low-grade serous carcinoma (LGSC), endometrioid ovarian carcinoma (ENOC), mucinous carcinoma (MC), and clear cell ovarian carcinoma (CCOC). Currently, primary screening methods for OC include histopathological examination, transvaginal ultrasound, and detection of tumor markers such as cancer antigen 125 (CA125) and human epididymis protein 4 (HE4). Survival rates in OC are strongly influenced by the stage at diagnosis, with a 5-year survival rate of approximately 20% in stage IV, 40% in stage III, 70% in stage II, and 90% in stage I (3). However, statistics show that less than 30% of patients are diagnosed at an early stage of OC, largely due to the limitations of current screening methods, which contribute to late diagnosis and poorer outcomes.

In recent years, the role of gut microbiome (GM) in cancer has received significant attention. The GM consists of bacteria, viruses, fungi, archaea, and protozoa in the digestive tract. It has been proven that GM plays a crucial role in maintaining host homeostasis and modulating disease development. The alteration in GM composition, known as dysbiosis, contributes to cancer pathogenesis through interrelated mechanisms involving chronic inflammation, immune modulation, altered estrogen metabolism and microbial metabolites production (4). Dysbiosis can compromise intestinal barrier integrity, allowing bacterial components such as lipopolysaccharides to enter systemic circulation and activate pattern recognition receptors, including Toll-like receptors, thereby triggering persistent inflammation that promotes cellular proliferation, angiogenesis and tumor progression (5, 6). Moreover, GM influences estrogen metabolism by increasing β-glucuronidase-producing bacteria, which enhance estrogen deconjugation and reabsorption, leading to elevated circulating estrogen levels that stimulate hormone-dependent tumor growth (7). Microbial metabolites also play dual roles in tumorigenesis, where secondary bile acids and lactate can promote DNA damage, pro-inflammatory signaling and metastasis, while short-chain fatty acids such as butyrate exhibit anti-inflammatory and tumor-suppressive effects (8, 9). Specific bacterial taxa, including *Fusobacterium nucleatum* and *Escherichia coli* have been implicated in promoting DNA damage, immune evasion and inflammatory responses (10, 11), whereas *Coriobacteriaceae* and *Bifidobacterium* have been associated with enhanced lactate metabolism, particularly in chemotherapy-resistant malignancies (12). In OC, elevated estrogen levels may bind to estrogen receptors on ovarian epithelial cells, promoting DNA synthesis, cell proliferation, and resistance to apoptosis. Oxidative estrogen metabolites can induce mutagenic DNA damage that facilitates malignant transformation (13).

Cancer patients often exhibit reduced gut bacterial diversity and abundance compared to healthy individuals, suggesting a link between gut dysbiosis and tumorigenesis. Specific bacterial taxa have been associated with the initiation and progression of various malignancies. For example, decreased levels of *Ruminococcus 2* in the GM have been correlated with cervical cancer (14). Meanwhile, excessive aggregation of *Clostridium nucleatum* has been associated with poor prognosis in metastatic colorectal cancer (CRC) (15). In endometrial cancer, *Ruminococcus* sp. N15.MGS-57 and C16:1 have been identified as potential biomarkers associated with distinct clinical features and outcomes (16). GM imbalance may promote tumorigenesis by altering metabolic functions and estrogen levels, thus influencing the onset and progression of endometrial cancer (17). Beyond carcinogenesis, the GM also modulates host responses to cancer therapy. In patients with metastatic melanoma treated with ipilimumab, enrichment of *Faecalibacterium* and other Firmicutes was associated with longer progression-free and overall survival, while members of the Bacteroidetes phylum were enriched in patients with colitis resistance (18).

Overall, these findings suggest that the GM may contribute to the development and progression of OC. However, the specific compositions and influence of GM in OC remain poorly understood, particularly regarding disease progression and treatment outcomes. Therefore, this systematic review aims to comprehensively integrate current evidence on alterations in the human microbiome associated with OC or EOC and its treatment outcomes, focusing on microbial composition and diversity. The review includes studies investigating GM alterations in OC broadly, as well as those focusing specifically on EOC, to provide a comprehensive understanding of microbial diversity and its potential associations with disease development and outcomes.

## 2 Methods

### 2.1 Search strategy

This systematic literature review complied with guidelines outlined by the Preferred Reported Items for Systematic Reviews and Meta-Analysis (PRISMA) (19). A comprehensive literature search was systematically carried out using the two electronic databases, PubMed and Web of Science, to identify all relevant articles related to GM and OC. Z.B., C.K.T., D.W., and M.N.S. discussed these search strategies for different databases. A systematic literature search was performed until 31st January 2024 and all data were pooled and kept in EndNote software (Version 20) software (Clarivate, UK). Detailed search string for each database, including Boolean operators, MeSH terms, and an applied filter, is presented in **Supplementary Table 1**. An additional search was conducted on 12 October 2025, using the same search strategy to include any recent articles. The search was limited to English-language articles involving human participants published up to October 2025.

### 2.2 Inclusion and exclusion criteria

Studies were included based on the following criteria: (i) original research article, (ii) studies on GM and OC, and (iii) human studies. However, studies were excluded if they met any of the following conditions: (i) gray literature included meeting abstracts, editorials, case reports, and review articles, (ii) the study did not involve the GM and OC, (iii) a lack of baseline characteristics data, and (iv) non-English articles or not available. This systematic review included studies focusing on either ovarian cancer in general or specifically on EOC, which is the most common histological subtype.

### 2.3 Screening of articles for eligibility

Z.B., C.K.T., and D.W. were involved in the data extraction process, with disagreements resolved by a fourth reviewer (M.N.S.). The manual screening of the reference lists of all included articles was conducted to enhance the robustness of the search strategy. After removing all duplicates, these articles’ titles and abstracts were reviewed based on inclusion and exclusion criteria. A data collection form was used to standardize the data collection, and each researcher performed all data extraction independently. Any disagreements were resolved through discussion, with final decisions based on majority consensus. Additionally, records of the reasons for excluding articles were documented for reference.

**Figure 1:**
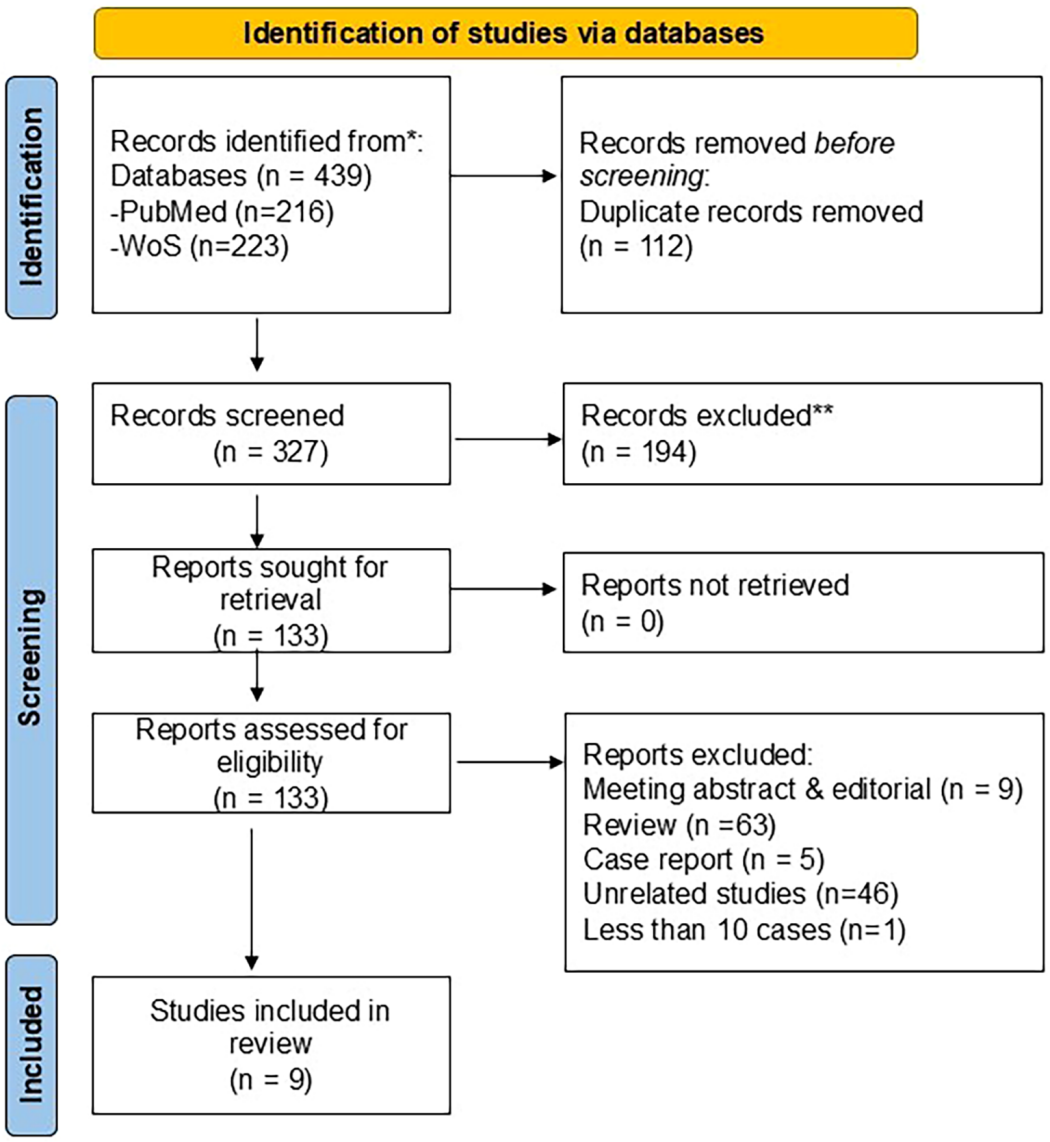
Flowchart of the process of literature search and extraction of studies meeting the inclusion criteria.

Our database search identified a total of 439 articles. After 112 duplicates had been removed, 327 articles were left for further screening. A total of 133 articles were chosen for a full-text screening. Following a full-text review, 124 articles were excluded for the following reasons: meeting abstracts and editorials (n = 9), review papers (n = 63), case reports (n = 5), unrelated studies (n = 46), and studies with fewer than 10 cases (n =1). Only nine studies (12, 20–27) met the inclusion criteria and were included in this systematic study. The study flowchart is illustrated in **Figure 1**.

### 2.4 Risk of bias assessment

The risk of bias was independently assessed by two authors (Z.B. and N.F.N.M.S.), with any disagreements consulted and resolved by a third reviewer (M.N.S). The Risk of Bias in Non-Randomized Studies of Exposure (ROBINS-E) tool was used to evaluate the included studies (28). This tool assessed bias across seven domains: (1) confounding, (2) selection of participants, (3) measurement of exposure, (4) post-exposure interventions, (5) missing data, (6) measurement of outcomes and (7) selection of the reported result. Each study was rated as “Low”, “Some concerns”, “High” or “Very high” risk of bias. The findings were visualized in a summary plot using the Risk-of-bias VISualization (robvis) (29) to provide a clear overview of the risk of bias in the included studies.

## 3 Results

### 3.1 Characteristics of reviewed studies

The extracted data from these studies include (1) author and publication year, (2) country of origin, (3) study design, (4) number of sample size, (5) detection method, (6) sample type, (7) time of intervention, and (8) reported GM alteration in OC patients, as summarized in **Table 1**. All nine studies were published between 2020 and 2025 and were conducted across multiple countries. This review includes cross-sectional (24–26), longitudinal (12), observational (27), retrospective (22) and case-control designs (23). All of these studies used 16S rRNA gene sequencing to profile microbiota in stool samples. Most of the articles included in this review investigated the association between the GM and OC, while only three studies specifically focused on EOC (12, 24, 25).

**Table 1:** Characteristics of the included studies.

Author (Year)	Cancer types	Country	Study design	Sample size	Detection method			Sample type	Time of intervention	Gut microbiota changes
Chen et al. (25) (2025)	EOC	China	Cross-sectional study	34 patients with EOC, 15 patients with benign ovarian tumor, 30 healthy volunteers	16S rRNA Sequencing			Stool	Diagnosis	+
D'Amico et al. (12) (2021)	EOC	Italy	Longitudinal study	24 OC patients, 24 controls	16S rRNA Sequencing			Stool	Chemotherapy	+
Gong et al. (23) (2021)	OC	China	Case-control study	77 chemo-resistant patients, 97 chemo-sensitive patients	16S rRNA Sequencing			Stool	Chemotherapy	+
Gong et al. (26) (2025)	OC	China	Cross-sectional study	148 OC patients, 91 benign ovarian tumor patients, 90 other benign tumor patients and 53 healthy controls	16S rRNA Sequencing			Stool	Chemotherapy	+
Hu et al. (24) (2023)	EOC	China	Cross-sectional study	20 EOC patients, 20 benign ovarian tumor patients, 20 healthy controls			16S rRNA Sequencing	Stool	Diagnosis	+
Jacobson et al. (22) (2021)	OC	United State	Retrospective study	40 OC patients, 5 controls	16S rRNA Sequencing			Stool	Chemotherapy	+
Okazawa-Sakai et al. (27) 2025	OC	Japan	Observational study	56 patients with OC • BRCA1/2mut-positive patients: 23 patients. • BRCA1/2mut-negative patients: 33 patients	16S rRNA Sequencing			Stool	PARP inhibitors (PARPi) treatment	+
Tong et al. (21) (2020)	OC	China	Longitudinal study	18 OC patients		16S rRNA Sequencing		Stool	Postoperative	+
									Chemotherapy	+
Wang et al. (20) (2022)	OC	China	Cross-sectional study	40 OC patients, 40 benign disease controls		16S rRNA Sequencing		Stool	Diagnosis	+

“+” indicates the presence of reported gut microbiota changes. OC: ovarian cancer; EOC: Epithelial ovarian cancer, PFI: Platinum-free interval; EBOT: epithelial benign ovarian tumor, PPR: Primary platinum-resistance.

Across all studies, notable GM alterations were reported. These changes were observed at different clinical time points, including at diagnosis, during the postoperative period, throughout or after chemotherapy and during PARP inhibitor therapy. Three studies specifically compared chemotherapy-sensitive and chemotherapy-resistant patients (12, 22, 23), while one study evaluated the association between GM composition and the efficacy of PARP inhibitors in patients with OC (27). The remaining studies investigated differences in GM composition between OC or EOC, benign tumors, and healthy controls (20, 21, 24–26).

### 3.2 Risk of bias studies

**Figure 2:**
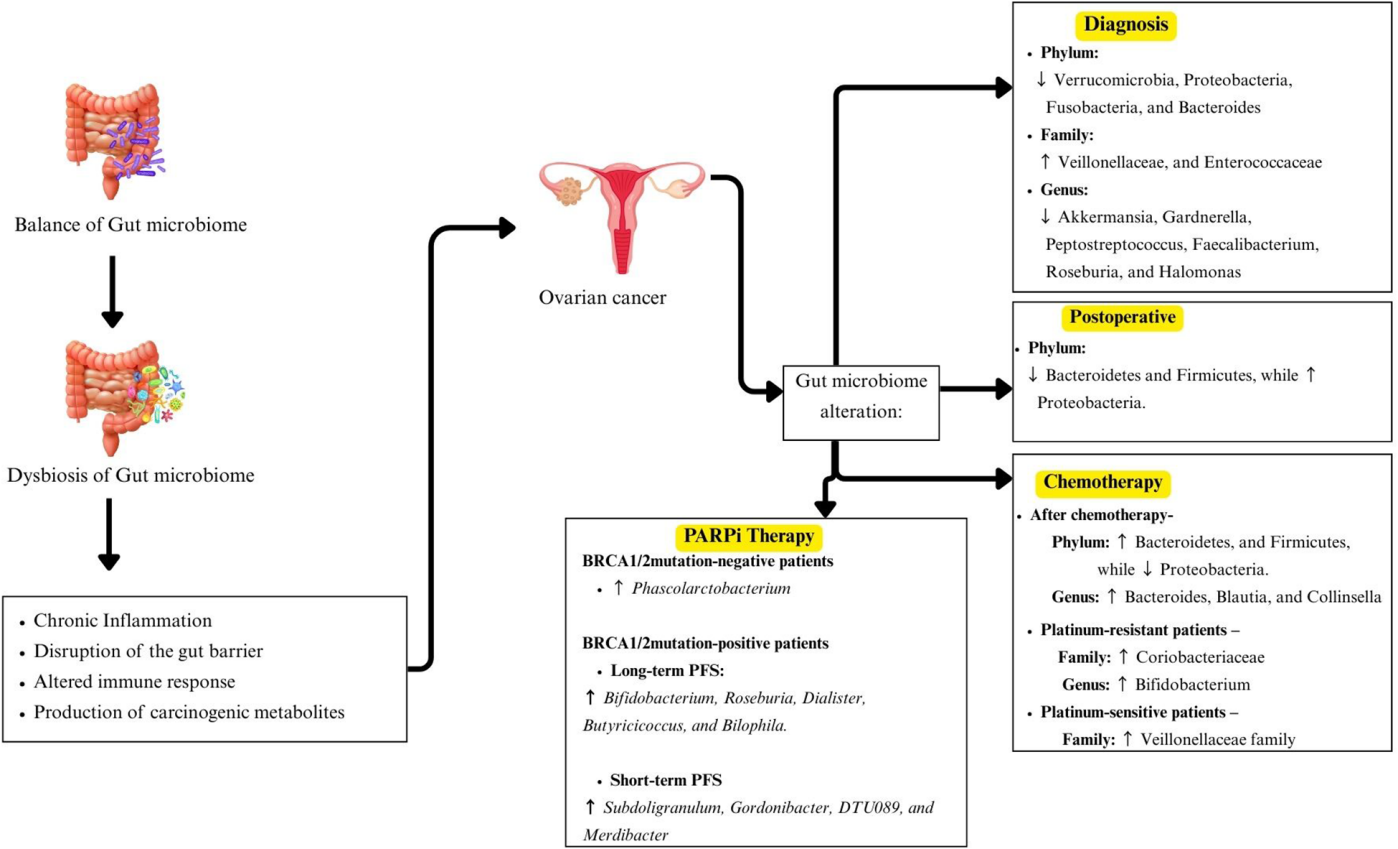
Result analysis for risk assessment using ROBINS-E tool.

The risk of bias for the included studies was assessed using the ROBINS-E tool, as shown in **Figure 2**. Most studies demonstrated a low to some concern risk of bias in the domains related to selection of participants, exposure classification, and missing data (**Supplementary Table 2**). However, high to very high risk of bias was observed in the domains of confounding and selection of reported results, particularly due to limited adjustment for key covariates, such as diet, antibiotic use or comorbidities. The measurement of the outcomes domain generally showed some concerns because blinding of outcome assessors was seldom reported. Overall, the included studies were rated as some concern to high risk, indicating that the results should be interpreted with caution.

### 3.3 GM compositions of OC at diagnosis

There was no significant difference in α-diversity between OC or EOC patients and healthy women before surgery, with both groups predominantly harboring Firmicutes, Bacteroidetes, Actinobacteria and, Proteobacteria (12, 20, 21, 24). However, β-diversity showed a significant difference between the two groups.

At the phylum level, EOC patients exhibited a significant increase in Proteobacteria, accompanied by a decrease in Firmicutes, Actinobacteria and Verrucomicrobia (20, 21, 24). In advanced EOC cases, the GM profile deviated further from healthy controls, with a more pronounced increase in Proteobacteria and a further decline in Actinobacteria (24). Conversely, Chen et al. (25) reported enrichment of Firmicutes, indicating possible compositional variation across cohorts. At the family level, EOC patients showed reduced abundances of Lachnospiraceae, Bifidobacteriaceae, Clostridiaceae, Rikenellaceae, and Porphyromonadaceae, while Coriobacteriaceae was significantly increased (12).

At the genus level, *Akkermansia*, the only genus within *Verrucomicrobia*, significantly declined in OC patients. Linear discriminant analysis (LDA) supported this finding, suggesting that the absence of *Akkermansia* may be a key microbial signature of OC (20). Additionally, EOC patients had increased levels of *Prevotella, Bacteroides, Adlercreutzia, Collinsella, Lactococcus*, *Lachnobacterium, Escherichia-Shigella* and tumor-associated genera such as *Flavonifractor, Ruminococcus-gnavus-*group and *Anaerotruncus*, whereas beneficial or probiotic genera including *Bifidobacterium, Ruminococcaceae_Ruminococcus, Coprococcus, Blautia, Dorea, Lachnospira*, *Roseburia*, and *Bifidobacterium* were either reduced or absent in EOC patients (22, 25, 26). At the species level, *Escherichia coli* was increased in EOC patients with the enrichment of opportunistic genera such as *Escherichia* and *Shigella* (25).

### 3.4 Perioperative changes in GM composition

Only one study has examined GM differences before and after radical surgery in OC patients (21). The study found significant differences in the intestinal microbiota at all taxonomic levels. At the phylum level, Firmicutes was the dominant phylum in preoperative fecal samples (Group B). However, in postoperative samples (Group M0), the dominant phylum shifted to Proteobacteria. Despite this shift, both Firmicutes and Proteobacteria showed a significant overall decrease post-surgery. Additionally, Actinobacteria exhibited a decrease after surgery, although the change was not statistically significant.

The relative abundance of several genera was markedly reduced in Group M0 compared to Group B, including *Bacteroides, Bilophila, Faecalibacterium, Collinsella*, and *Coprococcus.* Conversely, *Klebsiella, Enterobacter*, and E*nterococcu*s were significantly higher in post-surgery. Other genera such as *Lachnospiraceae_Ruminococcus, Blautia, Roseburia, Prevotella*, and *Collinsella* also showed decreased trends postoperatively, though these changes were not statistically significant.

To further explore these microbial shifts, Linear Discriminant Analysis Effect Size (LEfSe) analysis was employed. The analysis revealed that Firmicutes and Proteobacteria were dominant phyla in Group B and M0, respectively. At the genus level, *Bilophila* and *Faecalibacterium* were key genera of Group B, while *Klebsiella* and *Enterococcus* were predominant in Group M0.

### 3.5 GM compositions associated with targeted therapy (PARP inhibitor)

The composition of the GM may influence the efficacy of PARP inhibitor (PARPi) therapy in EOC patients, potentially affecting progression-free survival (PFS) in a BRCA mutation-dependent manner (27). A higher abundance of *Phascolartobacterium* was significantly associated with longer PFS in patients without BRCA1/2 mutation. In BRCA1/2 mutation-positive patients, long-term PFS correlated with increased levels of *Bifidobacterium*, *Roseburia, Dialister*, *Butyricoccus*, and *Bilophila*. Conversely, short-term PFS in this group was associated with higher abundance of *Subdoligranulum, Gordonibacter, DTU089* and *Merdibacter.***Table 2** displayed a summary of reported gut microbiota alterations observed in ovarian cancer patients across all included studies.

**Table 2:** Summary of reported gut microbiota alterations in ovarian cancer patients.

Author (Year)	Time of intervention	GM composition
Chen et al. (25) 2025	Diagnosis (EOC vs benign ovarian patients vs healthy control)	EOC patients – • Phylum: *↑* Firmicutes • Genus: *↑ Escherichia and Shigella.* * ↓ Bifidobacterium, Ruminococcus* and *Bilophila* • Species: *↑ Escherichia coli*
Gong et al. (26) 2025	Diagnosis (OC patients vs Benign ovarian patients vs other benign patients vs healthy control)	Ovarian Tumor vs. Healthy Controls • Genus: *↑ Escherichia_Shigella, Bacteroides and Prevotella. Proteobacteria, and Bacteroidetes* ↓*Coprococcus*, *Fusicatenibacter*, *Butyricicoccus*, and *Oscillibacter* Ovarian cancer vs. Benign Ovarian Tumor • Genus: *↑ Flavonifractor*, *Ruminococcus_gnavus_group*, and *Anaerotruncus Bacteroides* and *Escherichia_Shigella*. ↓ *Prevotella* and *Veillonella* Ovarian Tumor vs. Other Benign Tumor • Genus: * ↑Fusicatenibacter*, *Butyricicoccus*, *Coprococcus*, *Parasutterella*, and *Anaerotruncus*
Hu et al. (24) (2023)	Diagnosis (OC vs EBOT vs healthy controls)	Healthy control – • Phylum: *↑* Actinobacteria • Genus: *↑* Bifidobacterium, *Ruminococcaceae_Ruminococcus*, and *Collinsella* EOC group- • Phylum: *↑* Proteobacteria, Fusobacteria, and Bacteroides • Genus: * ↑ Gardnerella*, and *Peptostreptococcus* EBOT group- • Family: *↑* Veillonellaceae, and Enterococcaceae • Genus*: ↑ Faecalibacterium, Roseburia*, and *Halomonas*
Okazawa-Sakai et al. (27) 2025	PARPi Therapy (BRCA1/2mut-positive patients vs BRCA1/2mut-negative patients)	In BRCA1/2mutation-negative patients • *↑ Phascolarctobacterium* was significantly associated with longer PFS • In BRCA1/2mutation-positive patients • Long-term PFS: *↑ Bifidobacterium*, *Roseburia*, *Dialister*, *Butyricicoccus*, and *Bilophila*. • Short-term PFS * ↑ Subdoligranulum*, *Gordonibacter*, *DTU089*, and *Merdibacter*
Tong et al. (21) (2020)	Postoperative and Chemotherapy (Cycles M1-M5)	Postoperative- • Phylum: ↓ Bacteroidetes and Firmicutes, while ↑ Proteobacteria.
		After chemotherapy- • Phylum: ↑ Bacteroidetes, and Firmicutes, while ↓ Proteobacteria. • Genus: * ↑ Bacteroides, Blautia*, and *Collinsella*
Wang et al. (20) (2022)	Diagnosis (OC vs benign disease controls	OC patients: • Phylum: * ↓* Verrucomicrobia • Genus: ↓ *Akkermansia*

OC: ovarian cancer; PFI: Platinum-free interval; EBOT: epithelial benign ovarian tumor, PPR: Primary platinum-resistance, PFS: Progression-Free Survival. * ↑*: Increase and * ↓*: Decrease.

### 3.6 GM compositions associated with chemotherapy

In OC or EOC patients undergoing chemotherapy, the dominant bacterial phyla identified as Firmicutes, Proteobacteria, Bacteroidetes, Actinobacteria, and Verrucomicrobia (12, 21–23). Among these, Firmicutes consistently remained the most prevalent across different treatment stages (12, 21, 22). Chemotherapy impacted the relative abundance of several phyla, with some fluctuations observed across cycles. For example, in platinum-sensitive (PS) patients receiving neoadjuvant chemotherapy, members of Bacteroidetes, such as *Bacteroides, Prevotella*, and *Parabacteroides*, were notably enriched (12).

At the family level, significant changes were observed following chemotherapy. Coriobacteriaceae increased progressively during treatment, particularly in platinum-resistant (PR) patients, and was associated with lower survival probability. This family includes genera such as *Eggerthella* and *Collinsella.* In contrast, Veillonellaceae was more abundant in PS patients and linked to improved survival outcomes. Other key families affected by chemotherapy included Lachnospiraceae, Enterobacteriaceae, Bifidobacteriaceae, Lactobacillaceae, Erysipelotrichaceae, Ruminococcaceae, and Mogibacteriaceae, all of which showed treatment-related changes in relative abundance.

At the genus level, the dominant or frequently observed genera during chemotherapy included Lachnospiraceae_unclassified, Blautia, Ruminococcus, Clostridiaceae_Unclassified, Coriobacteriaceae, Enterobacteriaceae_unclassified, Bifidobacterium, Bacteroides, Faecalibacterium, Collinsella, Bilophila, Coprococcus, Klebsiella, Enterobacter, Enterococcus, Veillonella, Akkermansia, Prevotella, and Lactobacillaceae_unclassified (12, 21, 23). Chemotherapy led to a significant reduction in the relative abundance of certain genera, such as Enterobacteriaceae_unclassified, Klebsiella, and Enterobacter (21). Conversely, other genera, including Bacteroides, Blautia, Collinsella, Bilophila, Faecalibacterium, and Coprococcus showed increased abundance during treatment (12, 21, 23). Interestingly, some genera (Enterobacteriaceae_unclassified, Klebsiella, Enterobacter, Bilophila, Enterococcus, Coprococcus, Veillonella, Bifidobacterium, Akkermansia and Lactobacillaceae_unclassified) returned to near pre-chemotherapy levels over multiple treatment cycles (21).

Differences in GM composition were also observed between PR and PS patients in EOC (12). PR patients had significantly higher in Coriobacteriaceae, especially *Eggerthella* and *Bifidobacterium*, while PS patients exhibited increased levels of Veillonellaceae*, Catenibacterium*, and *Anaerotruncus* (12). These compositional shifts were associated with clinical outcomes, with higher Coriobacteriaceae levels correlating with reduced survival and higher Veillonellaceae levels with improved survival (12). The type of chemotherapy further influenced microbial profiles. In PS patients receiving adjuvant chemotherapy, enriched taxa included *Bacteroides, Prevotella*, and *Parabacteroides*, *Faecalibacterium*, and *Acidaminococcus*, while PS patients on neoadjuvant chemotherapy had higher levels of *Desulfovibrio, Paraprevotella, Anaerostipes, Sutterella*, and *Pseudoramibacter_Eubacterium*. Notably, in neoadjuvant-treated patients, Coriobacteriaceae abundance increased over time, while Ruminococcaceae, especially *Faecalibacterium* and *Ruminococcus*, decreased (12).

Longitudinal profiling using Spearman Correlation Analysis across five chemotherapy cycles (M1-5) revealed stage-specific dominant genera. Before chemotherapy, *Corynebacterium* and *Klebsiella* were dominant. During the cycles, *Erysipelotrichaceae_Unclassified* and *Ruminococcus* were most relevant in M1; *Clostridiaceae_Unclassified* and SMB53 in M2; *Lactococcus* and *Prevotella* in M3, *Prevotella* and *Rothia* in M4; and *Gemella* and *Mogibacteriaceae_Unclassified* in M5 (21). The correlation coefficients for these associations were higher (R values = 0.89 to 0.99), indicating strong temporal relationships. According to the random forest model identified *Angelakisella*, *Arenimonas*, and *Roseburia* were identified as the top three most important predictors associated with OC resistance (23). Chemotherapy-related alterations in GM composition and their clinical associations are summarized in **Table 3**.

**Table 3:** Summary of gut microbiota (GM) compositional changes and clinical associations in ovarian cancer patients following chemotherapy.

Author (Year)	Time of intervention	Group	GM composition	Clinical associations
D'Amico et al. (12) (2021)	Chemotherapy (Platinum-sensitive vs Platinum-resistant patients)	Platinum-resistant patients	• Alpha diversity: Reduced and remained low • Beta diversity: Greater temporal instability • Family: ↑ Coriobacteriaceae • Genus: ↑ *Bifidobacterium* and *Eggerthella*	Lower survival
		Platinum-sensitive patients	• Alpha diversity: Relatively stable and higher • Beta diversity: More stable over time • Family: ↑ Veillonellaceae	Longer survival
Gong et al. (23) (2021)	Chemotherapy (resistant vs non-resistant OC patients)	Chemo-sensitive OC patients	• Phylum: ↑ Firmicutes	Chemo-sensitive outcome
		Chemo-resistant OC patients	• ↑ Alpha diversity • Phylum: ↑ Proteobacteria • Genus: ↑*Angelakisella, Arenimonas, Roseburia*	Chemoresistance outcome
Jacobson et al. (22) (2021)	Chemotherapy (Platinum-free interval < 6 months vs ≥ 24 months)	Primary Platinum Resistance (PPR) (PFI < 6 months)	• ↓ Alpha diversity • Phylum: ↑ Bacteroidetes, Firmicutes, Proteobacteria • Genus: ↑ *Clostridiales: Lachnospira, Unidentified Ruminococceae genus* and *Subdoligranulum*	Long-term relationship between platinum-resistance and the gut microbiome
		Platinum Super-Sensitive (PS) (PFI > 24 months)	• ↑ Alpha diversity • Phylum: ↑ Bacteroidetes, Firmicutes, Proteobacteria	A more stable or less unique GM profile compared to the PPR group

OC: ovarian cancer; PFI: Platinum-free interval, PPR: Primary platinum-resistance. * ↑*: Increase and * ↓*: Decrease.

## 4 Discussion

### 4.1 GM compositions of OC at diagnosis

This systematic review identified nine original research articles that examined GM compositions in stool samples from OC or EOC patients. Despite a broadly similar composition of dominant microbial phyla between OC patients and healthy individuals, several key changes in features were consistently observed. Notably, a significant increase in the relative abundance of Proteobacteria and a marked decrease in Firmicutes were observed in OC patients. This indicates a clear shift in microbial equilibrium. Similar results are consistent with a meta-analysis by Pammi et al. (30), which analyzed 14 studies. They concluded that GM dysbiosis preceding necrotizing enterocolitis in preterm infants was characterized by increased relative abundances of Proteobacteria and a decreased relative abundance of Firmicute*s* and Bacteroidetes. Similarly, the stage and subtype of EOC appear to exhibit a significant impact on GM composition. This includes reduced microbial diversity, as indicated by lower alpha diversity indices, and compositional shifts such as an increased abundance of Proteobacteria and decreased levels of *Actinobacteria, Bifidobacterium*, and *Ruminococcaceae_Ruminococcus.*

Mechanistically, the overrepresentation of Proteobacteria, a phylum of Gram-negative bacterium that includes pathogenic genera like *Salmonella, helicobacter, Enterobacter* and *Escherichia* (31), has been strongly linked to immune dysregulation, and carcinogenesis (32, 33). Lipopolysaccharide (LPS) derived from Proteobacteria activates Toll-like receptor 4 (TLR4), triggering the TLR4/MyD88/NF-Кβ signaling cascade (34). These conditions can promote pro-inflammatory cytokine release (IL-1β, IL-6 and TNF-α), angiogenesis and tumor proliferation (35). Persistent TLR4 activation enhances immune evasion and epithelial-mesenchymal transition (EMT), processes associated with OC progression and metastasis (36). Increased Proteobacteria has also been linked to oxidative stress and production of reactive oxygen species (ROS), which can induce DNA damage and activate oncogenic pathways such as PI3K/Akt and STAT3, further emphasizing tumor-promoting conditions (37, 38). Elevated Proteobacteria levels have also been significantly associated with other malignancies, including thyroid cancer, as demonstrated in both a systematic review and a Mendelian randomization analysis (39). Overall, an elevated abundance of Proteobacteria may reflect GM imbalance and may be associated with disease development and progression.

In contrast, the decline of beneficial taxa such as *Akkermansia muciniphila*, a mucin-degrading bacterium from the phylum Verrucomicrobiota, may compromise intestinal integrity and immunological balance (40). *A. muciniphila* maintains mucosal barrier function and produces beneficial short-chain fatty acids (SCFAs), including acetate and propionate, which exhibit anti-inflammatory effects via G-protein-coupled receptor (GPR43/109A) activation and suppression in NF-Кβ signaling (41). Besides, *A. muciniphila* has been shown to exhibit anti-tumor effects by activating and interacting with dendritic cells, which trigger the production of interleukin-12 (IL-12) (42). This process promotes the activation of cytotoxic T lymphocytes (CD8^+^), thereby enhancing the body’s immune response against tumors. Clinical studies have further associated its increased abundance with improved metabolic health, reduced atherosclerotic risk (43), enhanced GM diversity during weight loss (44), and protection against liver injury (45). Hence, reduced *A. muciniphila* levels have been associated with decreased mucus layer thickness, increased intestinal permeability and endotoxin leakage (40). The endotoxin leakage is a conducive condition to systemic inflammation and a tumor-promoting microenvironment.

OC patients also showed lower levels of certain bacterial families, such as Lachnospiraceae and Clostridiaceae (from the Firmicutes phylum), and Rikenellaceae and Porphyromonadaceae (from the Bacteroidetes phylum). This differs from the usual pattern observed in healthy individuals, where Firmicutes and Bacteroidetes are the most dominant and balanced groups in the GM (46). The depletion of these taxa may reflect impaired SCFA biosynthesis particularly of butyrate. Butyrate acts as a histone deacetylase (HDAC) inhibitor, promoting tumor suppressor gene expression, cell cycle arrest and apoptosis in malignant cells. Therefore, the loss of butyrate-producing bacteria such as *Lachnospiraceae* and *Clostridiaceae* removes an essential anti-tumor regulatory mechanism (47). This disruption promotes a pro-oncogenic niche associated with low-grade inflammation and immune dysregulation.

In addition, Bifidobacteriaceae, a well-known probiotic family, also exhibited variation, while a surprising increase in *Bacteroides* was observed. In another study, *Bacteroides thetaiotaomicron* is one of the species of *Bacteroides* is recognized for its role in carbohydrate fermentation and mucin degradation (48). It can disrupt mucosal integrity when overgrown, particularly under antibiotic pressure, leading to bacterial translocation, and increasing inflammation as seen in -versus-host disease (49). Other species of the *Bacteroides* have been implicated in systemic inflammatory disease, including malaria (50) and polycystic ovary syndrome (*Bacteroides vulgatus*) (51). Furthermore, alterations in *Bacteroides* abundance may influence cytokine signaling, as seen in colorectal cancer, where an inverse correlation was noted between *Bacteroides* spp. and interleukin 9 (IL-9) levels (52). Hence, these findings indicate that the depletion of SCFA-producing taxa together with the enrichment of Bacteroides disrupts intestinal immune homeostasis and may contribute to the pro-inflammatory microenvironment that facilitates ovarian tumorigenesis.

An increased abundance of *Prevotella* was another notable finding in OC patients. This genus has been previously associated with non-small cell lung cancer (53) as well as pre-hypertension and hypertension (54). Although *Prevotella* is a commensal organism, it has pathogenic potential, especially in the context of female genital tract infections, including endometritis, bacterial vaginosis, and chorioamnionitis (55). Mechanistically, *Prevotella* overgrowth activates the TLR2/TLR4 signaling pathway, leading to the upregulation of pro-inflammatory cytokines such as interleukin-1β (IL-1β), interleukin-6 (IL-6), and interleukin-23 (IL-23) (56). Furthermore, the secretion of virulence factors including ammonia, hydrolases, and sialidase, enhances mucin degradation, bacterial adherence, and impairs host immune defenses (55). These immunopathogenic mechanisms suggest a potential role for *Prevotella* in promoting epithelial invasion, immune evasion and oncogenic inflammation within the female reproductive tract, thereby contributing to the pathophysiology of OC.

*Collinsella*, another genus elevated in OC patients, has shown similar patterns of dysbiosis in conditions such as autism spectrum disorders (57) and childhood-onset asthma (17). *Collinsella* has been associated with increased intestinal permeability and enhanced disease severity, as demonstrated in arthritis models (58). However, some evidence suggests a potentially beneficial role for *Collinsella aerofaciens*, which was found in higher abundance among responders to anti-PD 1-based immunotherapy in metastatic melanoma (59). Interestingly, *Collinsella* and other genera such as *Roseburia, Blautia*, and *Lachnospiraceae-unclassified* were found to be depleted in adolescent depression but restored following sertraline treatment, with *Roseburia* in particular exhibiting high predictive potential for therapeutic response (60). While *Roseburia is decreased in OC*, it was found to be enriched in NSCLC, further illustrating the disease-specific context of GM shifts (53).

Besides, the genus *Ruminococcus* was significantly reduced in OC patients, a trend similarly observed in individuals with major depressive disorder, infantile cholestasis and cardiovascular diseases (61). *Ruminococcu*s-dominated enterotypes are enriched in *Ruminococcus* and *Akkermansia*, play an important role in breaking down complex carbohydrates and supporting the gut’s protective barrier (62). Mechanistically, *Ruminococcus* species contribute to gut homeostasis through the fermentation of dietary polysaccharides and resistant starch into SCFAs, particularly butyrate (63). Butyrate serves as a primary source for colonocytes and enhances mucosal integrity. Moreover, *Ruminococcus* may facilitate mucin degradation and cross-feeding interactions with other commensals, thereby maintaining microbial diversity and epithelial health (64). Its depletion may be a signal of a loss of metabolic and structural elasticity within the gut ecosystem.

### 4.2 GM compositions of OC after surgery

There is growing evidence that surgical interventions have a significant impact on GM. In line with observations in OC, a study by Yu et al. (65) found that *Klebsiella* levels increased post-bariatric surgery, whereas beneficial genera such as *Bacteroides, Coprococcus*, and *Faecalibacterium* significantly declined. Moreover, they reported a notable rise in alpha diversity, particularly at the 3 months after surgery, accompanied by proliferation of *Streptococcus, Akkermansia*, and *Prevotella*. These genera are often associated with mucosal health and metabolic regulation.

In line with these findings, Özdemir et al. (66) observed a significant enhancement in GM alpha diversity in individuals with morbid obesity following bariatric surgery, eventually reaching levels similar to non-obese controls by 6 months post-operation. At the phylum level, there was a marked increase in Bacteroidetes and a concurrent reduction in Firmicutes, resulting in a significant reduction in the Firmicutes/Bacteroidetes ratio. This ratio is often associated with improved metabolic outcomes. Notably, genera such as *Lactobacillus* and *Bifidobacterium* decreased, while *Akkermansia*, a mucin-degrading bacterium linked to gut barrier integrity, significantly increased.

Ben Izhak et al. (67) also showed an increase in the phyla Proteobacteria and Fusobacteria abundance, and a decrease in Firmicutes after bariatric surgery. At the class level, Beta- and Gamma- proteobacteri*a* were dramatically elevated, underscoring a shift toward potentially pro-inflammatory microbial populations.

Beyond metabolic surgeries, other surgical procedures such as appendectomy have also been impacted in long-term GM alterations and disease risk. A 20-year longitudinal study by Shi et al. (68) revealed that individuals who underwent appendectomy had a 73% increased risk of developing colorectal cancer (CRC). This was accompanied by an enrichment of seven CRC-promoting bacteria, including *Bacteroides fragilis* and a reduction in five commensal species. Interestingly, *Fusobacterium nucleatum*, a well-known CRC-associated species, was decreased after radical surgery in CRC patients. However, *Clostridium scindens*, a bile acid-transforming species linked to carcinogenesis via production of deoxycholate (DCA), along with upregulation of its associated bile acid-inducing genes (bai operon) (69). These findings highlight the substantial and surgery-specific reshaping of the GM. Such shifts not only reflect physiological adaptation but may also influence postoperative outcomes, including immune response modulation, metabolic improvement, and even oncogenesis.

### 4.3 GM compositions associated with targeted therapy

The study identified a specific GM, *Phascolarctobacterium*, whose high baseline abundance was significantly associated with longer PFS in OC patients receiving PARPi maintenance therapy, especially among those who were BRCA1/2 mutation-negative (27). *Phascolarbacterium* is a commensal bacterium known to produce SCFAs, particularly propionate and acetate (70). These SCFAs can enhance CD8^+^ T cells (71), which aligns mechanistically with PARPi-induced activation of the DNA-sensing type 1 interferon pathway (71, 72). The presence of *Phascolarbacterium* may synergistically enhance immunostimulatory effects of PARPi therapy, improving tumor control and delaying progression. These findings highlight the potential utility of GM profiling as a predictive biomarker for PARPi response, particularly in BRCA1/2 mutation-negative patients. The consistent association of *Phascolarbacterium* abundance with favorable outcomes suggests that microbiome modulation through dietary strategies, probiotic supplementation or FMT may enhance therapeutic efficacy.

In contrast, among BRCA1/2 mutation-positive patients, Linear Discriminant Analysis Effect Size (LEfSe) identified several SCFA-producing genera, including *Bifidobacterium*, *Roseburia*, *Dialister*, *Butyricicoccus*, and *Bilophila*, that were enriched in long-term responders. These bacteria are known for their immunomodulatory and anti-inflammatory functions, which may support a favorable response to therapy (73, 74). Meanwhile, higher abundances of *Subdoligranulum*, *Gordonibacter*, *DTU089*, and *Merdibacter* were associated with shorter PFS, suggesting potential roles as resistance-associated taxa, although this requires further validation.

### 4.4 GM composition associated with chemotherapy

Although research on the impact of chemotherapy on the GM is limited, available evidence highlights its significant impact on GM composition and diversity. Post-chemotherapy changes often mirror microbial imbalances observed at diagnosis. For instance, elevated levels of *Bacteroides* and *Collinsella* were reported after chemotherapy, reflecting their resilience or proliferation in response to treatment-induced perturbations. Similarly, a study in CRC patients demonstrated an increase in *Bacteroides plebeius* following chemotherapy, while another study reported minimal changes in *Bacteroides* and *bifidobacteria* (75). In contrast, Stringer et al. (76) observed that chemotherapy-induced diarrhea in cancer patients was associated with reduced levels of *Bacteroides*, *Bifidobacterium*, *Lactobacillus*, and *Enterococcus*, alongside increased abundance of opportunistic pathogens such as *Escherichia coli* and *Staphylococcus* spp. reflecting a shift towards a dysbiosis state.

In OC, PS patients displayed reduced GM diversity, with a notable increase in Coriobacteriaceae, *Bifidobacterium*, and *Eggerthella* (12). Coriobacteriaceae and *Bifidobacterium* are known as lactate-producing bacteria (77). Elevated lactate levels contribute to tumor progression via the Warburg effect. This effect is a metabolic reprogramming in cancer cells characterized by aerobic glycolysis and lactate overproduction. This metabolic shift triggers tumor growth, immune evasion, angiogenesis, and metastasis and reduced chemotherapeutic efficacy (77). Furthermore, Coriobacteriaceae has been affected in the promotion of colorectal tumorigenesis in high-fat diet-fed mice and has shown enrichment in acute myeloid leukemia, tuberculosis, and carcinoid syndrome patients (78–80). Coriobacteriaceae correlates with elevated of lipid-associated markers such *as* hydroxypropyl-hydroxyproline, prolyltyrosine, tyrosyl-proline, total cholesterol, and low-density lipoprotein-1 (LDL-1), and low-density lipoprotein-2 (LDL-2) (79). Interestingly, a contradictory role was reported in allergic rhinitis, where Coriobacteriaceae showed a potential protective effect (81).

*Eggerthella*, another genus overrepresented in PS OC patients, has been consistently linked with various conditions, including psychiatric disorders (major depressive disorder, bipolar disorder, psychosis and schizophrenia), autoimmune disease (Crohn’s disease, ulcerative colitis and rheumatoid arthritis), and multiple sclerosis, reflecting its potential role in systemic inflammation and host-microbiome interactions (82).

In contrast, *Veillonellaceae* was found to be enriched in PS OC patients, suggesting a potentially beneficial role. This family comprises lactate-utilizing bacteria, which may reduce excess lactate accumulation in the tumor microenvironment. *Veillonella atypica*, for example, metabolizes lactate to propionate. Scheiman et al. (83) demonstrated that *V. atypica* supplementation improved athletic performance in marathon runners, and similar mechanisms may support host resilience during chemotherapy. However, increased levels of Veillonellaceae have also been associated with a higher intrahepatic cholangiocarcinoma.

Notably, both *Faecalibacterium* and *Ruminococcus* were key genera associated with gut barrier integrity and anti-inflammatory function and found to decrease in OC patients undergoing neoadjuvant chemotherapy (12). *Faecalibacterium prausnitzii*, in particular, is considered a hallmark of gut health, and its reduction has been linked to inflammatory diseases and postoperative Crohn’s disease recurrence. *In vitro* studies show that *Faecalibacterium prausnitzii* can suppress the production of proinflammatory cytokines such as interleukin-8 (IL-8) and inhibit the nuclear factor kappa beta (NF-Кβ) signaling pathway, thereby exerting immunomodulatory effects (84). However, a different species, *Fusobacterium nucleatum*, was found to be enriched in relapsed CRC patients post-chemotherapy. *Fusobacterium nucleatum* has been affected in chemoresistance by activating the autophagy pathway and modulating host responses through the TLR4-MYD88 axis and specific microRNA signaling, thus impacting therapeutic efficacy (85).

These microbial changes are closely linked with clinical parameters. Shorter survival correlates with higher *Bifidobacterium, Megamonas*, and *Pseudomonas*, whereas longer survival associates with lower *Klebsiella* and *Fusobacterium* abundance (21). Random forest modeling demonstrates that GM profiles can predict chemotherapy response with an AUC of 0.909, underscoring the prognostic potential of GM signatures (23). Furthermore, taxa such as *Ruminococcus, Desulfovibrio*, and *Lactobacillus* are associated with gastrointestinal adverse effects, highlighting the dual role of GM in therapeutic outcomes and patient quality of life (21).

The higher abundance of *Roseburia* from the Firmicutes phylum has emerged as a notable microbial signature in chemotherapy-resistant OC patients, even though *Roseburia* is typically regarded as a beneficial commensal that supports gut health. Mechanistically, *Roseburia* produces SCFAs, particularly butyrate, which modulate host immune responses by promoting regulatory T cell (Treg) expansion and exhibiting anti-inflammatory effects (73, 86). While these actions are generally protective, during cancer treatment, they might unintentionally weaken the body’s anti-tumor response. This can allow cancer cells to survive and make chemotherapy less effective. Metabolites produced by *Roseburia* can also affect cellular metabolism and oxidative stress, potentially reducing the levels of ROS that are crucial for chemotherapy-induced cancer cell death (87). Therefore, higher *Roseburia* levels in platinum-resistant patients may indicate a gut-driven immune and metabolic change that promotes resistance, highlighting its potential as a biomarker for treatment response and a target for microbiome-based therapy.

Overall, chemotherapy-induced shifts in GM are multifaceted and potentially bidirectional, either attenuating and exacerbating treatment outcomes depending on the microbial signatures involved.

### 4.5 Clinical significance between OC and GM

The observed changes in GM composition across disease stages and treatment phases highlight the potential clinical significance of the microbiome in OC. Specific taxa such as *Proteobacteria, Prevotella*, and *Collinsella*, which are enriched in OC, may serve as microbial signatures indicative of dysbiosis, systemic inflammation, and tumor-promoting conditions. In contrast, the reduction of beneficial taxa like *Akkermansia muciniphila*, *Faecalibacterium Prausnitzii*, and *Ruminococcus* underscores a loss of gut barrier integrity and anti-inflammatory capacity, which may influence disease progression and treatment outcomes.

These microbial shifts may have diagnostic and prognostic implications, as they could be developed into non-invasive biomarkers for early detection or treatment response. Besides, modulation of GM through probiotics, dietary intervention, or fecal microbiota transplantation (FMT) may present a promising therapeutic strategy to enhance chemotherapy efficacy, reduce toxicity and improve overall patient outcomes. Future longitudinal and interventional studies are warranted to validate these associations and to explore the translational potential of microbiome-targeted strategies in clinical oncology.

### 4.6 Limitations

This review has several limitations that should be considered. Firstly, individual differences in diet, genetic background and environmental exposure were not adequately addressed in the included studies. These factors are well-established modulators of microbial diversity and function and may have contributed to the heterogeneity of findings observed between studies. Their omission limits the ability to attribute microbiome alteration solely to OC or its treatment effects.

Secondly, this review included only nine studies, which is a relatively small number for a systematic review. Moreover, most studies had small sample sizes and were geographically concentrated, which may limit the generalizability of the findings. The small evidence base restricts the strength of conclusions that can be drawn and highlights the need for more well-designed, multi-center investigations in this field.

Thirdly, there was notable heterogeneity in sequencing platforms, bioinformatic pipelines, and analytical methods used across studies, which may have influenced the reported taxonomic and functional outcomes. Consequently, the certainty of evidence remains low, and current findings should be interpreted as hypothesis-generating rather than definitive.

Fourthly, the findings of this systematic review should be interpreted in light of the methodological limitations of the included studies. Several studies were rated as having some concerns to very high risks of bias, especially in participant selection, selection of reported result, confounding control, and outcome measurement. These biases may have affected the internal validity and overall strength of the synthesized evidence, potentially leading to over- or underestimation of associations between GM profiles and OC characteristics. Therefore, the conclusions should be interpreted with caution, and future research with more rigorous and standardized designs are needed to validate these findings.

Fifthly, these review protocols were not prospectively registered in a public database such as PROSPERO. Although every effort was made to conduct the review systematically and meticulously. We recognize this limitation and propose that future reviews register techniques in advance to improve transparency and minimize possible bias.

Finally, an important gap in the literature is the limited exploration of how GM composition may relate to disease prognosis, including its potential impacts on disease progression, treatment response, and long-term survival outcomes in OC patients. Given that specific microbiota profiles may influence tumor microenvironment modulation, immune response and systemic inflammation, this represents a promising avenue for future research aimed at identifying prognostic microbiome biomarkers and therapeutic targets.

## 5 Conclusion

**Figure 3:**
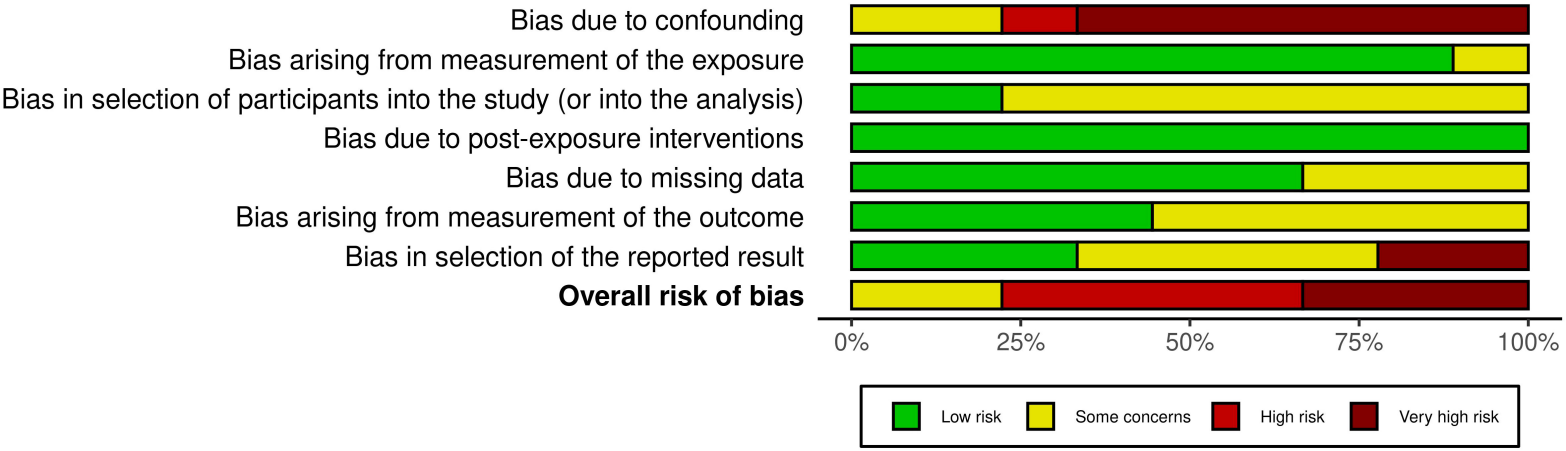
Summary of the association between gut microbiota and ovarian cancer.

In conclusion, this review highlights emerging evidence suggesting that alterations in GM composition may play a role in OC (**Figure 3**). Several studies report a relative increase in Proteobacteria and a reduction in *Akkermansia*, potentially indicating an association with disease risk and progression. However, these observations remain preliminary and inconclusive. Differences in GM profiles between OC patients and healthy individuals appear more distinct with advanced disease stages, and chemotherapy seems to induce notable shifts in microbial composition, with Firmicutes often becoming dominant. Some taxa, including *Angelakisella, Arenimonas* and *Roseburia*, have been proposed as potential biomarkers for future investigation. But their diagnostic or prognostic efficacy remains to be validated. Moreover, recent evidence indicates that a higher abundance of *Phascolartobacterium* is significantly associated with improved progression-free survival in BRCA1/2 mutation-negative patients undergoing PARPi therapy. This finding suggests that *Phascolarctobacterium* may serve as a promising predictive biomarker for treatment response, especially in guiding PARPi therapy.

These observations highlight the potential role of microbiome-targeted therapies, such as probiotics, prebiotics, or faucal microbiota transplantation, as complementary strategies in OC management. However, the evidence is currently limited by small sample sizes, methodological heterogeneity, and unaddressed confounding factors across studies. Further well-designed, longitudinal research is needed to clarify causal relationships, validate microbial biomarkers, and determine whether personalized microbiome-based intervention can improve treatment outcomes in OC patients.

## Data Availability

The original contributions presented in the study are included in the article/supplementary material. Further inquiries can be directed to the corresponding author.
