# Supplementary material for: Gut microbiome alterations and their clinical and biological implications in ovarian cancer: a systematic review
Source: Front Oncol. 2025 Nov 28;15:1690541. doi: 10.3389/fonc.2025.1690541 (PMC12698375; doi:10.3389/fonc.2025.1690541)
Supplement: Supplementary file 1 [file Table1.docx]

Supplementary Material

# Supplementary Data

Table Supplementary 1 Search strategies for different databases were used for the literature search

| **Database** | **Search string** | **Filter Applied** |
| --- | --- | --- |
| PubMed | (((“gut microbiota”[MeSH Terms]) OR (“gut microb*”[All Fields]) OR (“intestinal microb*”[ All Fields OR (“gut bacteri*”[ All Fields]) OR (“intestinal bacteri*”[ All Fields]))  AND  ((“ovarian neoplasms”[MeSH Terms]) OR (“ovarian cancer”[ All Fields]) OR (“ovarian carcinoma”[ All Fields]) OR (“carcinoma of ovary”[ All Fields]))) | - Language: English - Publication years: Up to October 2025 - Species: human |
| WoS | TS = ((“gut microb*” OR “intestinal microb*” OR “gut bacteri*” OR “intestinal bacteri*”) AND (“ovarian cancer” OR “ovarian carcinoma” OR “carcinoma of ovary”)) | - • Language: English  • Document type: Article  • Timespan: Up to October 2025 |

Abbreviation used depends on the database: TS: Topic search

# Table Supplementary 2 Risk of bias evaluation

| Study | D1 | D2 | D3 | D4 | D5 | D6 | D7 | Overall | |
| --- | --- | --- | --- | --- | --- | --- | --- | --- | --- |
| Chen at al. (2025) | Very high | Low | Some concerns | Low | Low | Some concerns | Very high | Very high |  |
| D'Amico et al. (2021) | Very high | Low | Some concerns | Low | Some concerns | Low | Low | High |  |
| Gong et al. (2021) | Very high | Low | Some concerns | Low | Low | Some concerns | Very high | Very high |  |
| Gong et al. (2025) | High | Low | Some concerns | Low | Low | Some concerns | Some concerns | High |  |
| Hu et al. (2023) | Very high | Some concerns | Some concerns | Low | Low | Some concerns | Some concerns | Very high |  |
| Jacobson et al. (2021) | Very high | Low | Some concerns | Low | Some concerns | Low | Low | High |  |
| Okazawa-Sakai et al. (2025) | Some concerns | Low | Low | Low | Low | Low | Some concerns | Some concerns |  |
| Tong et al., (2020) | Very high | Low | Some concerns | Low | Some concerns | Low | Low | High |  |
| Wang et al. (2022) | Some concerns | Low | Low | Low | Low | Some concerns | Some concerns | Some concerns |  |

# D1: Bias due to confounding; D2: Bias arising from measurement of the exposure; D3: Bias due to selection of participants; D4: Bias due to post exposure intervention post exposure intervention; D5: Bias due to missing data; D6: Bias in measurement of outcomes; B7: Bias in selection of the reported result.
